# Evaluation of the In-Vitro Effects of Albendazole, Mebendazole, and Praziquantel Nanocapsules against Protoscolices of Hydatid Cyst

**DOI:** 10.3390/pathogens13090790

**Published:** 2024-09-12

**Authors:** Nooshinmehr Soleymani, Soheil Sadr, Cinzia Santucciu, Abbas Rahdar, Giovanna Masala, Hassan Borji

**Affiliations:** 1 Department of Pathobiology, Faculty of Veterinary Medicine, Ferdowsi University of Mashhad, Mashhad P.O. Box 9177948974, Iran; nooshinmehrsoleymani@gmail.com (N.S.); soheil.sadr42@gmail.com (S.S.); 2 WOAH and NRL for Echinococcosis, Animal Health, Istituto Zooprofilattico Sperimentale della Sardegna, 07100 Sassari, Italy; giovanna.masala@izs-sardegna.it; 3 Department of Physics, University of Zabol, Zabol P.O. Box 538-98615, Iran

**Keywords:** hydatid cysts, nanocapsules, albendazole, mebendazole, praziquantel

## Abstract

Cystic echinococcosis still remains a serious health and economic problem worldwide. The etiologic agent is *Echinococcus granulosus sensu lato*, giving origin to a fluid-filled cystic lesion. Therapy faces several challenges. Nanodrugs have shown promise as chemotherapeutics against hydatid cysts. The present study evaluated a highly safe lipid nano-polymeric capsule for its superior efficacy and ability to overcome drug resistance. Nanocapsule drugs were formulated into six groups: Albendazole, mebendazole, praziquantel, albendazole + mebendazole, albendazole + praziquantel, and praziquantel + mebendazole. The protoscolicidal effects of these six groups were assessed at 10, 60, and 120 min in three concentrations (1, 0.5, and 0.25 mg/mL). Drug formulations were evaluated via zeta potential, droplet size, solubility, particle size analyzer (PSA), and scanning electron microscopy. According to the PSA results, the mean size of the albendazole nanocapsules was 193.01 nm, mebendazole was 170.40 nm, and praziquantel was 180.44 nm. Albendazole + mebendazole showed the greatest protoscolicidal activity at a concentration of 1 mg/mL after 120 min. In contrast, each drug’s 0.25 mg/mL single-dose times showed the least protoscolicidal activity after 120 min. With the right application of nanotechnology, it is possible to produce safe and effective drugs, such as the polymeric combination of albendazole and mebendazole, which has promising implications.

## 1. Introduction

The infections caused by metacestodes, the larval stage of *Echinococcus* species, can be asymptomatic for years or lead to severe illness and even death in animals and humans [1]. The species, representing a serious worldwide health problem and requiring priority veterinary and medical attention, are represented by *Echinococcus granulosus sensu lato* (*s.l*.) and *Echinococcus multilocularis*, the causative agents of cystic echinococcosis (CE) and alveolar echinococcosis (AE). These two taeniid tapeworms have different geographic distributions, pathogenicity, host specificity, and clinical symptoms [2,3].

Their biological cycles involve definitive hosts, particularly canids that may release eggs into the environment by their feces, and intermediate hosts, mainly rodents for *E. multilocularis* [4] and sheep or other ruminants for *E. granulosus s.l*. [4,5].

Researchers in the Middle East and Iran focus mainly on researching *E. granulosus s.l.*, since this species most often causes CE [6,7]. This region has a high incidence of *E. granulosus s.l*. infections due to its widespread presence in livestock and domestic dogs [8]. Understanding and controlling this particular species is crucial for developing public health efforts in these regions.

In detail, *E. granulosus s.l*.’s life cycle includes different stages in different hosts. This parasite first lives as an adult in the intestines of the definitive host, carnivores such as dogs [6]. Parasite eggs are excreted through the feces of these animals and enter the environment. Intermediate hosts, usually herbivorous animals, such as sheep and goats, contract hydatid cysts by eating grass contaminated with parasite eggs [7]. Cystic echinococcosis infections in humans are uncommon and can occur after eating infected raw food not properly washed [8,9,10] and contaminated by the eggs previously released into the environment. The eggs hatch in the intestines of these animals, and the larvae pass through the intestines [11]. After diffusion through the intestinal mucosa into the lymphatic system or the bloodstream, the parasite can spread into the organs, mainly the liver (65%) and lungs (25%), and seldom (10%) in other tissues (spleen, kidneys, heart, bones, and central nervous system), and give origin to a fluid-filled cystic lesion [12]. The larvae develop into hydatid cysts in these organs that can remain in the intermediate host’s body for years [13]. The parasite’s life cycle is completed when a carnivore consumes the infected flesh or viscera of the intermediate host, and the adult parasites settle in their intestine [14].

Cystic echinococcosis can be diagnosed and followed-up using imaging techniques, abdominal ultrasound (US), and chest radiography for an initial screening, in addition to magnetic resonance imaging (MRI) and computed tomography (CT) to detect more details of the lesion, such as vascularization and organization of adjacent organs, and to obtain a deeper diagnostic picture mostly necessary before surgical removal of the lesion [15]. Radiological investigations need to be supported by immunological analyses and useful tools capable of detecting the IgG antibodies anti-*Echinococcus*, such as immunochromatographic tests (ICTs), enzyme-linked immunosorbent assays (ELISAs), and immunoblotting (IB) [16,17].

The World Health Organization—Informal Working Group on Echinococcosis (WHO-IWGE) guidelines describe several medical treatments for managing patients affected by CE [18]. Surgery is the most effective treatment [17], but at the same time, it is very invasive, and if it is not properly performed, a new infection may arise as a major surgical complication [19]. Percutaneous therapy (PAIR) is a less deep procedure, but it does not comprise the removal of the echinococcal cyst. Moreover, the watch-and-wait (W/W) protocol is less invasive but requires strict follow-up. All treatments must be chosen according to the lesion’s characteristics (number, stadium, localization, etc.) [18]. However, all treatments described above must be put aside in the patient’s pharmacological management.

There are currently chemotherapeutic drugs that can treat CE with good results, including albendazole (ABZ), mebendazole (MBZ), praziquantel (PZQ), and benzimidazole (BMZ) [20,21]. Usually, the more-indicated drug for the management of CE is ABZ [21], prescribed in association with high-fat meals to increase bioavailability. But, sometimes, if this drug is unavailable, intolerable, or has side effects during the pharmacological treatment of CE patients, it is exchanged with another according to the guidelines [22]. It is known that BMZ side effects are common when high doses are administered for extended periods to human patients [23,24]. ABZ and MBZ have wide-spectrum anthelmintic activities by inhibiting the polymerization of a structural protein, tubulin, in the parasite’s intestinal cells. As a result, the microtubules cannot be constructed, and the life processes of the helminthic cells are interrupted [20]. PZQ acts causing spastic muscle paralysis and Ca^2+^ influx in the parasite. The worms are thus completely destroyed in the intestine or eliminated through the feces. Some BMZ compounds inhibit the biosynthesis of ergosterol required in the cell membranes of fungi and protozoa [25].

Recently, nanodrugs have emerged as potential pharmaceutical game-changers, significantly transforming therapeutic interventions [24]. It has been proven that nanodrugs can be used successfully to treat hydatid cysts [26,27,28,29]. Nanodrugs have demonstrated great promise as chemotherapeutics in hydatid cysts [30,31,32,33,34,35]. However, these nanomedicines have challenges that must be addressed and resolved before they can revolutionize treatment for hydatid cysts [32]. Nanotechnology must be harnessed for medical advancement as researchers balance therapeutic efficacy, safety profiles, and environmental impact [36,37,38]. The unique properties of nanodrugs make them ideal candidates for improving therapeutic outcomes in various medical conditions since they are small and have specific surface properties [39,40,41]. Nevertheless, there are challenges associated with using nanomedicine to treat hydatid cysts. A prominent concern, for example, is renal toxicity, in which nanoparticles can accumulate in the kidneys and cause adverse effects [42,43]. Moreover, the long-term effects of these nanoparticles on ecosystems are unknown, especially in terms of their composition and excretion [44,45,46]. The complex interactions between nanodrugs’ physicochemical properties, biodistribution patterns, and potential harm to patients and the environment necessitate a comprehensive approach that analyzes their therapeutic potential, biodistribution rates, and potential toxicity issues.

In the present study, nanocapsule systems have been explored as a potential solution to treat hydatid cysts with drug resistance [47,48] and to set new standards for safety, effectiveness, and sustainability. The present study aims to revolutionize how hydatid cysts are treated to bring about a new era of treatment that prioritizes patient well-being and environmental sustainability.

## 2. Materials and Methods

### 2.1. Collection of Protoscolices

Hydatid cysts were obtained aseptically from CE-infected sheep, and a 30-min sedimentation time allowed the hydatid fluid to settle in a cylinder for protoscolex isolation. After three rounds of washing with phosphate buffer saline (PBS), the protoscolices were recognizable as the settled bottom layer. Muscle movements and eosin staining were used for the viability assessment. The viability of protoscolices was assured through their movement under a light microscope, using the eosin exclusion test with a concentration of 0.1% eosin stain (1 g of eosin powder in 1000 mL distilled water). After 5 min of exposure, the unstained protoscolices were deemed viable, while stained protoscolices were considered dead. When 90% or more viable protoscolices were recorded in the sediments, the sample was appropriate for the experiments [49].

### 2.2. Development of Encapsulated Drugs

At room temperature, a suitably diluted amount of sodium caprylate (fatty acid) and PBS (pH 7.4) were vigorously mixed at a consistent ratio, and drug-to-pluronic and oil-to-surfactant molar ratios were determined in the form of microemulsions containing drugs incorporated in oil-in-water microemulsions which had been developed [50].

### 2.3. Zeta Potential, Particle Size Analyzer (PSA) Technique, and Scanning Electron Microscopy (SEM)

Twenty-four hours before the device examined the samples, ABZ solutions were made with lipid nano-polymeric capsules according to the instructions. Then, each prepared sample was poured into separate microtubes, mixed with 10 µL of PBS, and transferred to the central laboratory. The particle size and size distribution of the nanoparticles obtained were assessed using a particle size analyzer (PSA; SHIMADZU, SALD-2101, Tokyo, Japan). Additionally, the measurements were reconfirmed at predetermined intervals. Zeta potential was determined using Zeta-Chek (Microtract, ZC007, Haan/Duesseldor, Germany). Furthermore, for conducting scanning electron microscopy (SEM), the samples were first prepared using the Sputter Coater (SC7620) Polaron Range, and finally, SEM was performed by LEO 1450VP, 35 kV, Oberkochen, Germany.

### 2.4. Scolicidal Assay

A total of six nanocapsules were used in the study, ABZ, MBZ, PZQ, ABZ + MBZ, ABZ + PZQ, and PZQ + MBZ. At three times (10 min, 60 min, and 120 min), three concentrations (1, 0.5, and 0.25 mg/mL) were evaluated for their protoscolicidal effects. The sumac extract solution was prepared by dissolving dried extract in distilled water at 10, 30, and 50 mg/mL concentrations. A drop of protoscolex-rich sediment was combined with 2.5 mL of each sumac solution. Incubation was conducted at 37 °C for 10, 60, and 120 min after the contents had been gently mixed. Protoscolices were stained with 0.1% eosin after each incubation period after the upper phase had been carefully removed. Smeared protoscolices were examined under a light microscope after an incubation period of 15 min. At least 500 specimens were counted in each experiment to determine the percentage of dead protoscolices, with non-treated protoscolices as the control group. To ensure the accuracy of the results, the entire procedure was repeated three times.

### 2.5. Statistical Analysis

**Figure 1:**
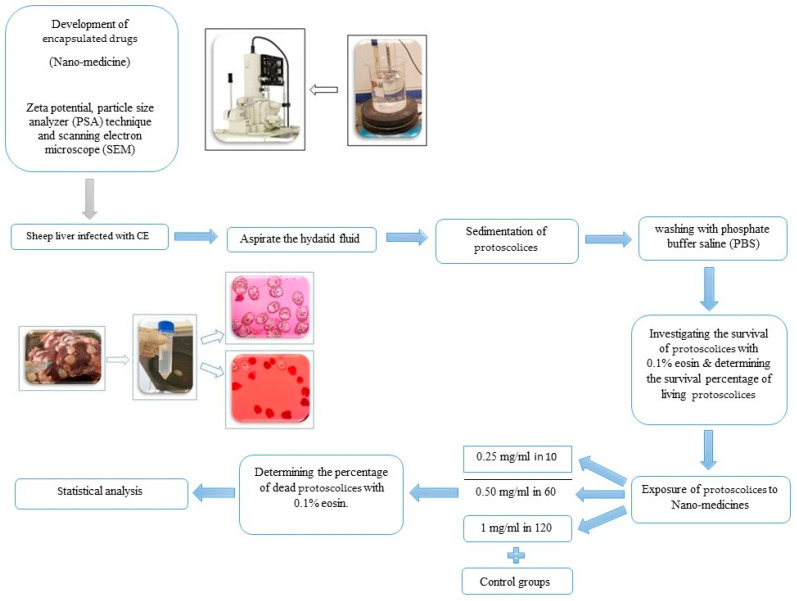
The schematic of the methodology in the current study.

As a result of this analytical approach, significant differences in protoscolicidal effects were identified among the different drug classes and concentrations. R version 4.3.1 (16 June 2023)—“Beagle Scouts” Copyright© 2023 The R Foundation for Statistical Computing Platform: aarch64-apple-darwin20 (64-bit) was used for statistical analysis. The data from this study were compared using the Kruskal–Wallis test. The Mann–Whitney test and *t*-test were used to compare between groups. Furthermore, post hoc tests included Bonferroni and Tukey–Kramer. The statistical tests were conducted with significance levels of less than 0.05%. The schematic of the methodology in the current study is illustrated in Figure 1.

## 3. Results

### 3.1. Zeta Potential, PSA, and SEM Results

**Figure 2:**
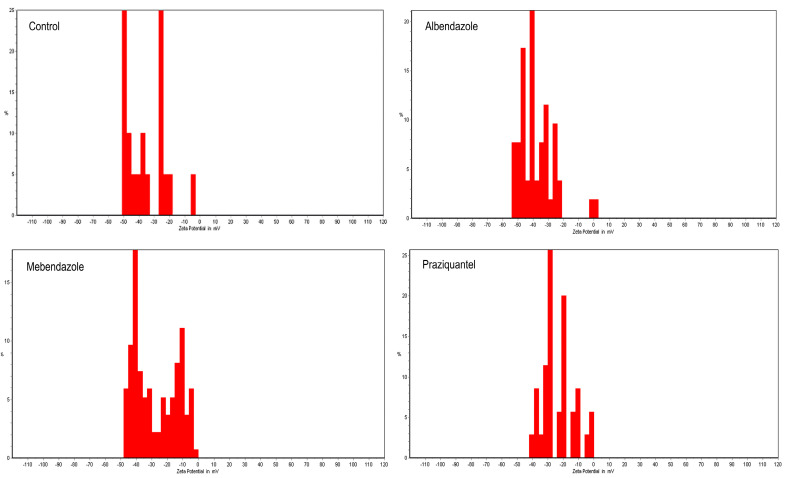
Zeta potential spectra of the nanocapsules: control, loaded with ABZ range of 5 to −55 millivolts (mean: −35.78 mW), MBZ range of 0 to −48 millivolts (mean: −27.38 mW), and PZQ range of 0 to −43 millivolts (mean: −23.34 mW).

**Figure 3:**
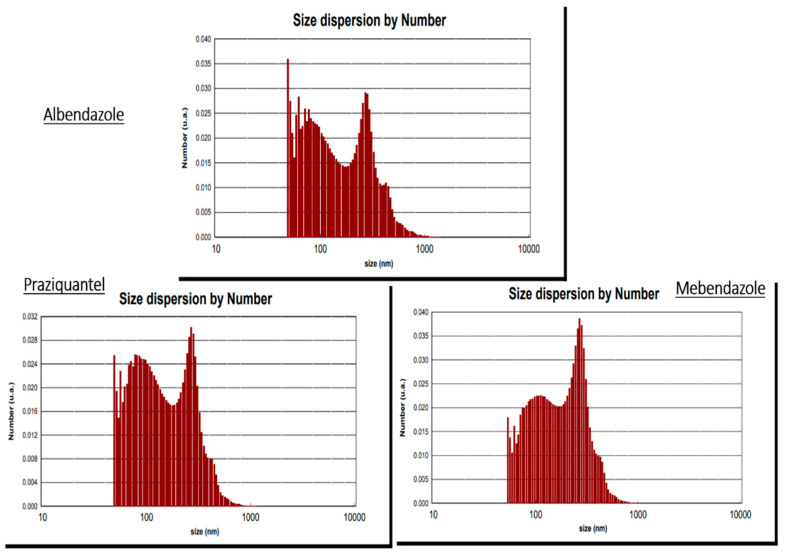
The mean size of ABZ nanocapsules is 193.01 nm, MBZ is 170.40 nm, and PZQ is 180.44 nm.

**Figure 4:**
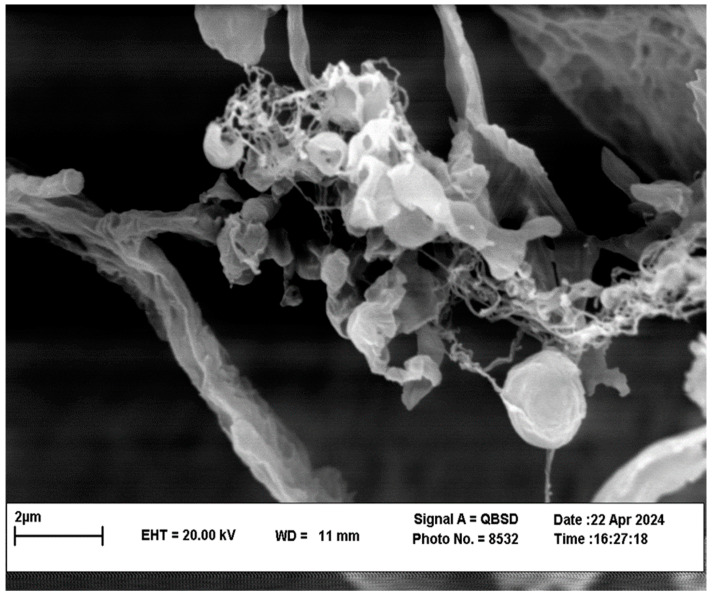
SEM image of polymeric nanocapsules.

The zeta potential spectra of the ABZ, MBZ, and PZQ are shown in Figure 2. It can be concluded in the graphs that the nanocapsules loaded with ABZ are in the range of 5 to −55 millivolts (mean: −35.78 mW), MBZ is in the range of 0 to −48 millivolts (mean: −27.38 mW), and PZQ is in the range of 0 to −43 millivolts (mean: −23.34 mW). A higher percentage of electric charges are present and have more peaks than the graph of free drugs, which shows that the stabilization of the drug on the nanoparticles has been completed well. They have higher stability in the environment than the drug. According to the PSA results, the mean size of the ABZ nanocapsules was 193.01 nm, MBZ was 170.40 nm, and PZQ was 180.44 nm (Figure 3). Figure 4 shows the morphology of the polymeric nanocapsules. The figure shows that the polymeric nanocapsules are sphere-like and of different sizes (Figure 4).

### 3.2. Protoscolicidal Rate

**Figure 5:**
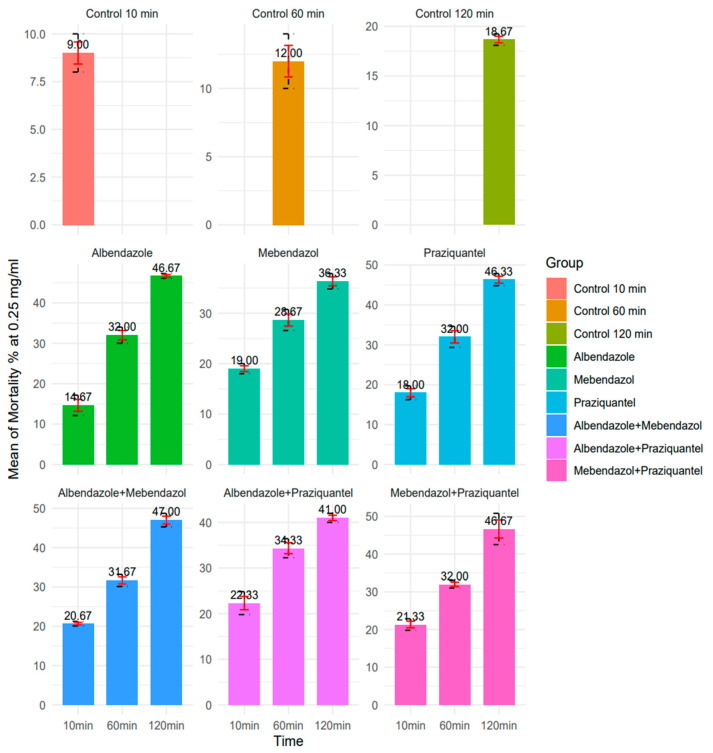
The protoscolicidal rate of 0.25 mg/mL concentration nanocapsules in 10, 60, and 120 min (R version 4.3.1 used to generate the graphs).

In 10, 60, and 120 min, the 0.25 mg/mL ABZ concentration exhibited respective protoscolicidal rates of 14.6%, 32%, and 46.6%. For MBZ, the protoscolicidal rate was 19%, 28.6%, and 36.3%. Additionally, the protoscolicidal rate for PZQ was 18%, 32%, and 46.33%, respectively. Furthermore, in 10, 60, and 120 min, at a 0.25 mg/mL concentration for ABZ + MBZ, the protoscolicidal rate was 20.6%, 31.6%, and 47%. For ABZ + PZQ, the protoscolicidal rate was 22.33%, 34.3%, and 41%. Moreover, MBZ + PZQ’s protoscolicidal rate was 21.3%, 32%, and 46.6%. Among these, PZQ exhibited the best performance at 120 min; albeit, all drugs showed significantly higher lethality percentages compared to the control group across all times (*p* < 0.05) (Figure 5).

**Figure 6:**
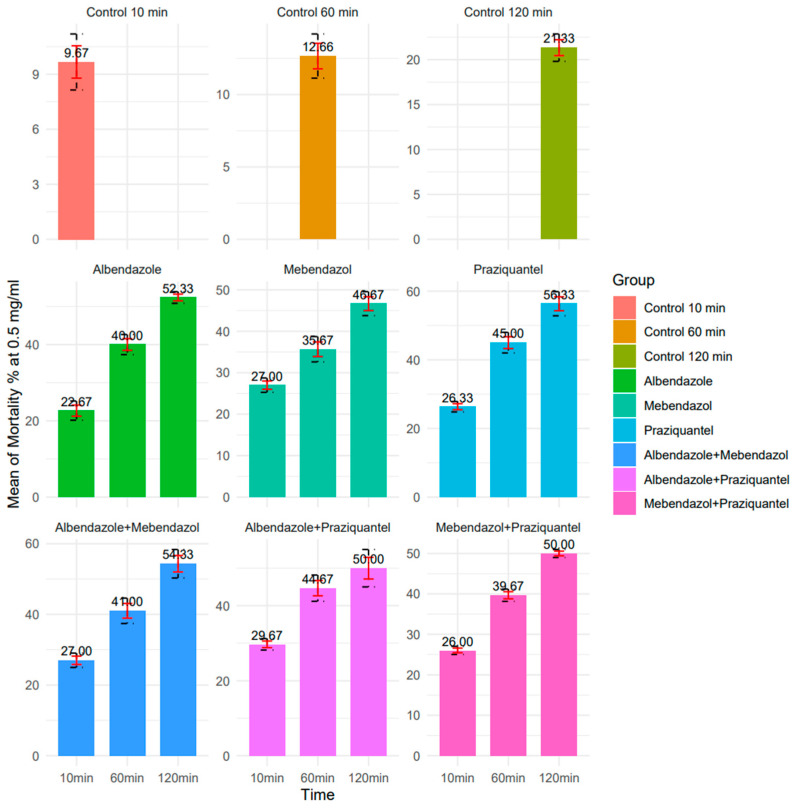
The protoscolicidal rate of 0.5 mg/mL concentration nanocapsules in 10, 60, and 120 min (R version 4.3.1 used to generate the graphs).

**Figure 7:**
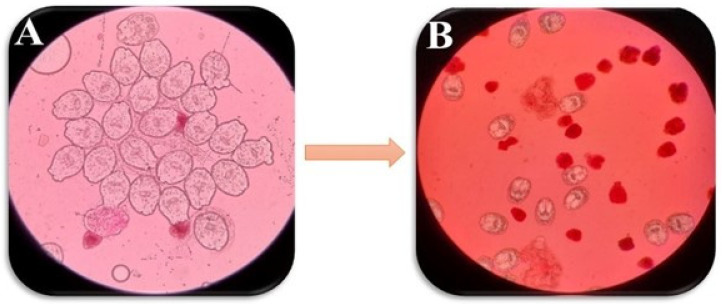
Light microscopy images showing the viable protoscolices of *Echinococcus granulosus* (stained with 0.1% eosin) (**A**: ×40). Live and red stained dead protoscolices after exposure to nano-ABZ (0.5 mg/mL) after 60 min of exposure (stained with 0.1% eosin) (**B**: ×10).

Regarding the 0.5 mg/mL concentration, in 10, 60, and 120 min, ABZ exhibited respective protoscolicidal rates of 22.6%, 40%, and 52.3%. For MBZ, the protoscolicidal rate was 27%, 35.6%, and 46.6%. Additionally, the protoscolicidal rate for PZQ was 26.3%, 45%, and 56.3%, respectively. Furthermore, in 10, 60, and 120 min, for the 0.5 mg/mL concentration of ABZ + MBZ, the protoscolicidal rate was 27%, 41%, and 54.3%. For ABZ + PZQ, the protoscolicidal rate was 29.6%, 44.6%, and 50%. Moreover, the MBZ + PZQ protoscolicidal rate was 26%, 39.6%, and 50%. Among these, ABZ exhibited the best performance at 120 min; albeit, all drugs showed significantly higher lethality percentages compared to the control group across all time intervals (*p* < 0.05) (Figure 6 and Figure 7).

**Figure 8:**
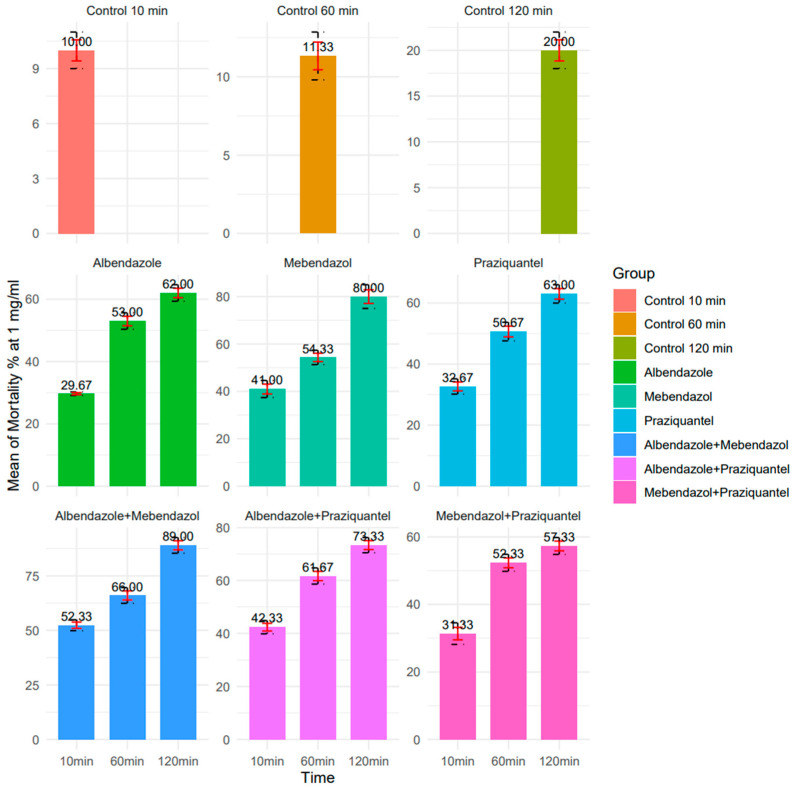
The protoscolicidal rate of 1 mg/mL concentration nanocapsules in 10, 60, and 120 min (R version 4.3.1 used to generate the graphs).

**Figure 9:**
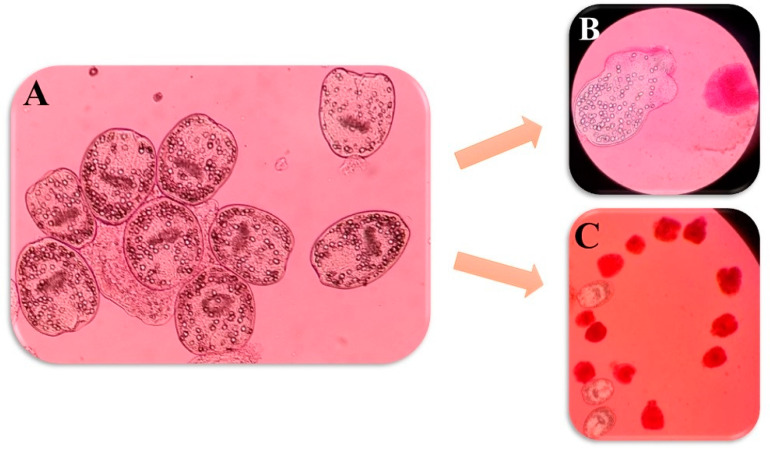
Light microscopy images showing the viable protoscolices of *Echinococcus granulosus* (**A**: ×40). Live and red stained dead protoscolices after exposure to nano-ABZ (1 mg/mL) after 10 min of exposure (stained with 0.1% eosin) (**B**: ×40, **C**: ×10).

Regarding 1 mg/mL concentration, in 10, 60, and 120 min, ABZ exhibited respective protoscolicidal rates of 29.6%, 53%, and 62%. For MBZ, the protoscolicidal rate was 41%, 54.3%, and 80%. Additionally, the protoscolicidal rate for PZQ was 32.6%, 50.6%, and 63%, respectively. Furthermore, in 10, 60, and 120 min, at a 1 mg/mL concentration for ABZ + MBZ, the protoscolicidal rate was 52.3%, 66%, and 89%. For ABZ + PZQ, the protoscolicidal rate was 42.3%, 61.6%, and 73.3%. Moreover, the MBZ + PZQ protoscolicidal rate was 31.3%, 52.3%, and 57.3%. Among these, ABZ + MBZ exhibited the best performance at 120 min; albeit, all drugs showed significantly higher lethality percentages compared to the control group across all time intervals (*p* < 0.05) (Figure 8 and Figure 9).

## 4. Discussion

Various parts of the world continue to be affected by cystic echinococcosis. Every single clinical management strategy of a patient, similarly to chemical treatment, is influenced by the hydatid cyst’s structure, localization, and stage. The host’s body produces a fibrous layer surrounding the cyst [51]. A monolayer of germinal cells encircles the parasite’s capsule in a 50 mm thick acellular complex consisting of mucopolysaccharides and proteins [52]. In the cyst fluid or on the germinal layer, these cells differentiate into protoscolices, which stay attached to these germinal layers [53]. During a cyst rupture or surgery, protoscolices can form new cysts if spilled on the intermediate host [19,54]. During cystogenesis, host and parasite proteins are present in a hypotonic fluid surrounding the cyst [55]. Therefore, active transport, selective absorption, and excretion must be possible through the cyst wall [52,56]. To kill the parasite tissues, drugs must penetrate all three layers of the cyst [57].

While chemotherapy has several potential benefits, it also has disadvantages [58,59,60]. There is considerable concern regarding drug resistance among parasites, where they evolve to withstand the effects of drugs to survive [61,62,63]. It is also important to note that the side effects of these chemotherapeutic agents can be highly damaging and significantly impact the patient’s quality of life [64]. There are several benefits associated with this procedure, as well as several drawbacks that necessitate the development of more effective and targeted treatment strategies [65].

The challenge is exacerbated because hydatid cysts are prevalent in many endemic regions where cystic echinococcosis is prominent. As a result, there is a significant barrier to managing chronic conditions due to limited access to healthcare facilities and resources [66]. A further burden created by the time it takes for patients to receive treatment for chronic infections is that it is both costly and challenging for healthcare systems to handle [67,68]. Thus, to address these multifaceted challenges, it is crucial to find innovative solutions that can deal with them. There is no doubt that in recent years, nanotechnology has emerged as one of the most potent forces to deal with hydatid cysts, more so than ever before [69,70]. With its exceptional physical and chemical properties, a nanoparticle can change treatment possibilities by adding a new dimension to them [71]. The ability of nanoparticles to deliver drugs with exceptional precision is one of their significant advantages, one of the most essential advantages of nanoparticles [72].

Albendazole is one of the main chemotherapy drugs used for the treatment of hydatid cysts, and it is used due to its good ability to kill parasites and reduce the size of cysts [73]. By disrupting the parasite’s glucose metabolism, this drug causes its death and destruction, and for this reason, it is very effective and widely used. However, there have been reports of some parasite strains developing resistance to albendazole, reducing its therapeutic efficacy and creating new treatment challenges. In this regard, nanotechnology and nano methods have played a very important role in enhancing the effects of albendazole [74,75]. Nanotechnology has significantly increased the efficiency of albendazole by improving the drug’s absorption capacity, increasing its stability and release time in the body, reducing side effects, and preventing parasite resistance. These achievements show the importance of using nanotechnology in improving chemotherapy treatments and increasing the effectiveness of drugs in dealing with parasitic diseases such as CE [76,77].

Liposomal nanoparticles can encapsulate therapeutic agents within lipid bilayers [78]. Encapsulating the drug in a protective film protects it from degradation so that it can be delivered directly to the cysts without any delays [79,80]. In this way, the therapeutic effect is enhanced and systemic toxicity is reduced, resulting in a better outcome. At the same time, nanotechnology offers extraordinary promise but must be cautiously approached to maximize its potential [81,82]. It is important to remember that nanotoxicity is a potential concern that cannot be ignored [83,84,85]. A lack of thorough study and understanding of nanoparticles can lead to their accumulation in vital organs and trigger unforeseen immune responses. Comprehensive toxicity assessments must be performed before these technologies are incorporated into clinical practice safely and effectively. Lipid microemulsion nanocapsules have emerged as one of the most promising nanotechnological breakthroughs among recent nanotechnology advancements [86]. Developing nanocapsules capable of being solubilized and stable and providing a controlled release of the drug offers an innovative solution to these problems. Researchers are experimenting with the use of nanocapsules to entrap antiparasitic medications to overcome the obstacles posed by drug resistance and the insufficient distribution of these drugs [87,88]. Using nanocapsules, drugs can be released over a prolonged period, preventing pharmaceutical concentrations from falling below therapeutic levels over time and potentially reducing the frequency of drug administration [89].

The presented data on the zeta potential spectrum of ABZ, MBZ, and PZQ nanocapsules reveal intriguing insights into the stability of these drugs when loaded onto nanocapsules. Notably, the higher stability observed in the environment for drug-loaded nanoparticles suggests an effective and improved stabilization process. This finding is crucial as it implies that the nanocapsules could potentially enhance the stability and efficacy of these drugs, offering a promising avenue for drug delivery systems [90,91,92]. Moving on to the protoscolicidal rates at different concentrations and time intervals, the results provide compelling evidence of the efficacy of ABZ, MBZ, and PZQ, individually and in combination. At a 0.25 mg/mL concentration, all three drugs and their combinations exhibited protoscolicidal rates significantly higher than the control group across different time intervals. Interestingly, PZQ demonstrated the best performance at 120 min. The trend continued at higher concentrations (0.5 mg/mL and 1 mg/mL), where the combined formulations, particularly ABZ + MBZ, consistently outperformed individual drugs regarding protoscolicidal rates. These findings underscore the potential synergistic effects of drug combinations and highlight the significance of nanocapsule-based drug delivery systems in enhancing the therapeutic outcomes of these anthelmintic drugs.

Using chitosan–ABZ (ChABZ) and chitosan–PZQ (ChPZQ) nanoparticles in both in vitro and in vivo experiments, researchers assessed the efficacy of ChABZ and ChPZQ in treating hydatid cysts [93]. A wide range of ChABZ and ChPZQ nanoparticle concentrations were used to treat microcysts formed in culture with either ABZ alone or in combination, as well as ABZ + PZQ suspensions and the combination of the two. An in vitro test demonstrated that combining ChABZ and ChPZQ nanoparticles showed significantly higher efficacy than ABZ + PZQ in combination. During prophylactic experiments in which ChABZ + ChPZQ nanoparticles were applied to infected mice, the amount and size of cysts were significantly reduced compared to those treated with the control group.

An investigation conducted by Fateh et al. in 2021 examined whether ABZ nanocrystals impacted the viability of *E. granulosus s.s*. protoscolices [94]. The nanocrystals had an average size and hydrodynamic diameter of 976 ± 218 and 1334 ± 502 nm, respectively. Using both morphological and molecular techniques, fertile hydatid cysts were obtained from slaughtered sheep’s livers, and those cysts were identified as belonging to *E. granulosus* s.s. based on their 100% similarity in morphology and genotype. They were tested on a small sample size to ascertain whether the protoscolices were susceptible to ABZ nanocrystals. There was a significant reduction in protoscolex viability due to the ingestion of 1 µg/mL nanocrystals and ABZ within 17 and 23 days, respectively. This indicates ABZ nanocrystals’ effectiveness in eliminating protoscolices.

According to a previous study, nanoformulations of ABZ were studied on protoscolices in vitro and in vivo to assess how they affected them [95]. Solid lipid nanoparticles containing ABZ were prepared using the microemulsion method. As a result of the successful storage of protoscolices in RPMI 1640 for one week, the survival of protoscolices was examined on days 3 and 7 of the experiment by testing the effect of ABZ at both 250 and 500 μg/mL concentrations. Protoscolices were injected into mice on day 3 at 250 μg/mL concentration to ascertain their pathogenicity. After three months, an autopsy was performed on the mice to assess their pathogenicity. Both ABZ and nano-ABZ showed the highest protoscolicidal efficacy on day 7 at both concentrations.

Moreover, researchers developed ABZ-loaded nanostructured lipid carriers (ABZ-NLCs) using a hot high-speed homogenization method [96]. This method increased the therapeutic efficiency of ABZ against *E granulosus* protoscolices and metacestodes. Methylene blue exclusion testing, scanning, and transmission electron microscopy were used to monitor the in vitro treatment of protoscolices and microcysts with free ABZ and ABZ–NLCs. An intraperitoneal injection of viable protoscolices was performed one day before administering chemoprophylactic treatment to Balb/C mice. After mice were treated with drugs intragastrically every day for 30 days, cyst size and frequency were evaluated to determine how effective the prophylactic treatment was. ABZ-NLCs were added to all protoscolices after 18 days of incubation, and the protoscolices were killed. On the other hand, 51.25% of those incubated in ABZ at 10 g/mL remained viable after 18 days. Between the ABZ-NLC group and the free ABZ group, hydatid cysts recovered from the ABZ–NLC group showed statistically significant differences in mean weight (*p* < 0.05), which resulted in a prophylactic efficacy of 92.45% and 38.53%, respectively. Germinal layer ultrastructural changes were observed in cysts treated with ABZ-NLCs.

In Table 1 is summarized the in vitro studies on the effects of nanomedicines against hydatid disease in recent years (Table 1).

Although innovative approaches can be successful, implementing them is challenging [110]. Alternative treatments, including surgery and cyst inactivation methods, are important. Surgery, one of the most effective treatments for CE, physically removes the cyst and yields health improvements in the patient, preventing serious complications. However, cyst inactivation methods can help supplement medical and surgical treatments and improve results [111].

It cannot be overstated that the accurate identification of species and genotypes is a cornerstone in treating CE. The potential for different species and genotypes to exhibit varied responses to treatments underscores the importance of this aspect. While this issue was not the focus of our present research, it is a crucial consideration for future studies. Identifying these parameters can significantly enhance treatment outcomes and enable more targeted treatments. Previous studies in this region of Iran [112,113] have indicated a high probability of the G1 genotype, further underscoring the importance of this aspect in our research.

Understanding the complex interactions between drug encapsulation, release kinetics, and immune interactions requires extensive research and development. Rigorous testing is necessary to ensure our nanotechnologies’ safety, effectiveness, and long-lasting benefits. This testing in both in vitro and in vivo models guarantees that our proposed nanotechnologies are thoroughly vetted and ready for implementation.

## 5. Conclusions

This study showed that nanocapsules of albendazole, mebendazole, and praziquantel have an effect on hydatid cyst protoscolices and are significantly better than the classic formulations of these drugs. Moreover, by controlling the release and increasing the absorption of the drugs, these nanocapsules strengthen their antiparasitic effects and reduce possible side effects. Therefore, nanocapsules can provide a more effective solution for cystic echinococcosis treatment and significantly improve existing treatments’ therapeutic efficiency and safety. Finally, the greatest protoscolicidal activity was evidenced by the albendazole + mebendazole nanocapsules at a concentration of 1 mg/mL after 120 min. The benefits of nanotechnology must be balanced with the risks associated with nanotoxicity, and it is essential to take a cautious approach. Cystic echinococcosis management could be revolutionized by integrating nanocapsules as research and development in this area progress.

## Figures and Tables

**Table 1:** In vitro studies on the effects of nanomedicines against CE in recent years.

Nanomedicine	Duration	Findings	Dosage	References
Cobalt oxide (CO)	5, 10, 15, 30, 60 min	CO: -Killed 100% of protoscolices of sheep liver cyst at 0.50 mg/mL in 60 min -Killed 100% of protoscolices of cattle liver cyst at 0.100 in 60 min -Killed 100% of protoscolices in goat liver cyst at 0.100 mg/mL in 30 and 60 min	0.025, 0.050, 0.100 (mg/mL)	Mahmoud, M.S. et al., 2023 [97]
Iron oxide nanoparticles coated with PO (FOMNPsP)	10, 20, 30, 60 min	FOMNPsP: Killed 100% of protoscolices -at 400 µg/mL after 10 min -at 200 and 300 µg/mL after 20 min -at 100 µg/mL after 30 min	100–400 μg/mL	Raziani, Y. et al., 2023 [98]
Curcumin nanoemulsion (CUR-NE)	10, 20, 30, 60, 120 min	CUR-NE: Killed 94% and 73.33% of protoscolices at 1250 and 625 (µg/mL) in 60 min	156, 312, 625, 1250 (µg/mL)	Teimouri, A. et al., 2023 [99]
Selenium nanoparticles (Se-NPs)	10, 20, 30, 60 min	Se-NPs: Killed 100% of protoscolices at 500 µg/mL after 20 min	50,100, 150, 200, 250, 350, 500 (µg/mL)	Mohammed, M.A. et al., 2022 [100]
Zinc nanoparticles (Zn-NPs)	5–60 min	Zn-NPs: Killed 81.6% of protoscolices at 200 μg/mL Zn-NPs + ALZ: Killed 100% of protoscolices at 200 μg/mL after 10 min	50, 100, 200 (μg/mL) Zn-NPs alone and combined with albendazole (100 μg/mL)	Shakibaie, M. et al., 2022 [28]
Biosynthesized zinc oxide nanoparticles (ZnO NPs) by Mentha longifolia L. leaves	15, 30 min and 1, 1.5, 2, 2.5 h	ZnO NPs: Killed 100% of protoscolices at 400 ppm in 150 min	100, 200, 400 ppm	Shnawa., B.H. et al., 2022 [101]
Copper nanoparticles (CuNPs)	5–60 min	CuNPs: Killed 73.3% of protoscolices at 750 mg/mL after 60 min	250, 500, 750 mg/mL	Ezzatkhah, F. et al., 2021 [31]
Silver–copper (core-shell) nanoparticles	10, 30, 60 min	Concentrations 250 and 500 mg/mL are the most effective in the vitality of the protoscolices compared to 50 and 125 mg/mL	50, 125, 250, 500 mg/mL	Aljanabi, A.A. et al., 2021 [102]
Zirconium oxide (ZrO_2_)	15, 30, 60 min.	ZrO_2_: Killed 53.1% of protoscolices at 4000 μg/mL in 60 min	250, 500, 1000, 2000, 4000 μg/mL	Abdul, A. and Ibrahim, J., 2020 [103]
-Piper nigrum (PN), -Ziziphus Spina-Christi (ZSC), -Eucalyptus globulus (EUCGLO) + Ag-NPs.	15, 30, 45 min	Killed more than 90% of protoscolices in minimum time.	1:10 2:10 3:10 (plants: Ag-NPs)	Salih, T.A. et al., 2020 [104]
Nanostructured lipid carriers of ivermectin	15, 30, 60, 120, 150 min	NLC-loaded IVM: Killed 100% of protoscolices at 400 and 800 μg/mL after 60 and 120 min	50, 100, 200, 400, 800 μg/mL	Ahmadpour, E. et al., 2019 [105]
Chitosan–praziquantel (ChPZQ) and chitosan–albendazole (ChABZ)	Microcyst culture flasks for 16 days	There were no viable microcysts observed on day 10 post-incubation with either 5 or 10 μg/mL doses of ChABZ + ChPZQ combination	1, 5, 10 μg/mL	Torabi, N. et al., 2018 [93]
Gold nanoparticles	5, 10, 20, 30, 60 min	Gold nanoparticles: Killed 76% of protoscolices at 4000 μg/mL in 60 min	250, 500, 1000, 2000, 4000 μg/mL	Napooni, S. et al., 2018 [106]
-Albendazole sulfoxide -ABZ-loaded PLGA-PEG	5, 10, 20, 30, 60 min	-ABZs: Killed 100% of protoscolices at 200 μg/mL in 20, 30, and 60 min -ABZ-loaded PLGA-PEG: Killed 100% of protoscolices at 150 and 200 μg/mL in all exposure times (5 to 60 min)	50, 100, 150, 200 (μg/mL)	Naseri, M. et al., 2016 [107]
Selenium nanoparticles (Se-NPs)	10, 20, 30, 60 min	Se-NPs: Killed 100% of protoscolices at 250 mg/mL in 20 min	50,100.150, 200, 250, 300, 350, 400, 450, 500 mg/mL	Mahmoudvand, H. et al., 2014 [108]
Albendazole sulfoxide, albendazole sulfone, and combined solutions	5 and 10 min	ABZ sulfone: 97.3%; ABZ sulfoxide: 98.4%; the combined solution: 98.6% were effective in 5 min	50 μg/mL	Adas, G. et al., 2009 [109]

## Data Availability

The datasets generated during the current study are available from the corresponding author upon reasonable request.
